# Dual-wavelength fiber-optic technique to assist needle cricothyroidotomy

**DOI:** 10.1007/s10103-020-03065-4

**Published:** 2020-07-23

**Authors:** Chien-Ching Lee, Chia-Chun Chuang, Bo-Cheng Lai, Chin-Li Lu, Edmund Cheung So, Bor-Shyh Lin

**Affiliations:** 1 Institute of Imaging and Biomedical Photonics, National Chiao Tung University, Tainan, 711 Taiwan; 2 Department of Anesthesia, An Nan Hospital, China Medical University, Tainan, Taiwan; 3 Department of Medical Sciences Industry, Chang Jung Christian University, Tainan, Taiwan; 4 Graduate Institute of Food Safety, College of Agriculture and Natural Resources, National Chung Hsing University, Taichung, Taiwan; 5 Graduate Institute of Medical Sciences, Chang Jung Christian University, Tainan, Taiwan

**Keywords:** Fiber optics, Airway, Cricothyroidotomy, Optical density, Hemoglobin parameter

## Abstract

The traditional needle cricothyroidotomy procedure is performed blindly without any medical equipment. Complications including posterior tracheal wall perforation, accidental vessel puncture, and missed tracheal puncture are reported. Therefore, we proposed a dual-wavelength fiber-optic technique based on the technique of near-infrared spectroscopy to assist operators performing needle cricothyroidotomy in a swine model. We embedded optical fibers in a 16-gauge intravenous needle catheter. Real-time data were displayed on an oscilloscope, and we used the program to analyze the data immediately. The change of optical density corresponding to 690-nm and 850-nm wavelengths and hemoglobin parameters (HbO_2_ and Hb concentrations) was analyzed immediately using the program in the laptop. Unique and significant optical differences were presented in this experiment. We could easily identify every different tissue by the change of optical density corresponding to 690-nm and 850-nm wavelengths and hemoglobin parameters (HbO_2_ and Hb concentrations). Statistical method (Kruskal-Wallis *H* test) was used to compare differences in tissues at each time-point, respectively. The *p* values in every tissue in optical density change corresponding to 690 nm and 850 nm were all < 0.001. Furthermore, the *p* values in every tissue in Hb and HbO_2_ were also all < 0.001. The results were statistically significant. This is the first and novel study to introduce a dual-wavelength embedded fibers into a standard cricothyroidotomy needle. This proposed system might be helpful to provide us real-time information of the advanced needle tip to decrease possible complications.

## Introduction

Management of the difficult airway is usually a challenge, and it is a very important issue for physicians [1]. In some emergency situations, inserting an intravenous needle catheter through the cricothyroid membrane is a lifesaving strategy that temporarily provides ventilation in the “cannot intubate, cannot ventilate” scenario [2–5]. It can provide the patient oxygen for only a temporary period, until a definitive airway is established. Performing the procedure of needle cricothyroidotomy is simple, and the equipment needed (16 gauge BD Angiocath™ IV Catheter) is readily available in clinical settings [6–8]. However, the conventional method is performed blindly, which can make it difficult to confirm needle location in some cases [9]. It is difficult to distinguish the tissue layers that the needle travels through, and it may cause damage to surrounding tissues. Reports of complications included vessel puncture, perforation of the posterior tracheal wall, barotrauma, or surgical emphysema when the prescribed technique is employed [10]. Although ultrasound-guided percutaneous tracheal puncture decreases these complications, it cannot be perspective to know the advancing needle tip under ultrasonographic guidance in an out-of-plane configuration [11, 12]. In the in-plane technique of ultrasound, the needle is parallel and directly under the probe. It permits the operators to see the entire needle shaft. Orthogonally, the needle is perpendicular to the probe in the out-of-plane technique. Operators only see a few millimeters of the needle shaft at once. It is controversial to choose the in-plane or out-of-plane technique when operators perform needle cricothyroidotomy procedure.

In recent years, fiber-optic techniques are developing and also applied to localize the tissue structure. The accurate and appropriate needle guidance to procedural targets is very crucial during percutaneous interventional procedures. Feng et al. displayed a method that a 0.9-mm microimaging fiber was delivered into a 16-gauge needle to develop a visual puncture system for performing needle cricothyroidotomy [13]. However, image resolution was so poor to identify airway structure and we could not identify every tissue layer (skin, fat, muscle, cartilage, cricothyroid membrane, and vessels) when the fiber was advanced into the trachea. Huang et al. presented a technique of using fiber-optic confocal microscopy for cardiac tissue discrimination. Their findings facilitated clinical translation of the fiber-optic confocal microscopy as an intraoperative imaging modality to reduce the incidence of conduction disturbances during surgical correction of congenital heart disease [14].

However, there is no suitable equipment for catheter cricothyroidotomy or needle cricothyroidotomy. In order to improve the above issues, a novel tracheal recognition system through cricothyroid membrane was proposed to specifically recognize different tissue layers. The proposed system could provide the near-infrared light source emitted from the optic fiber bundles contained within a 16-gauge intravenous needle catheter. By using the unique and significant optical differences, different tissues could be recognized. This system is applied for the technology of near-infrared spectroscopy to recognize the different tissues. In the theory of the near-infrared spectroscopy, the light irradiates into the tissue, and the reflected light changes with different tissues significantly, because different tissues have different absorption characteristics. Using this feature, the kind of tissue measured can be deduced. With the aid of this technology, we can guide the needle tip into the trachea easily.

## Materials and methods

### Study design and setting

**Fig. 1:**
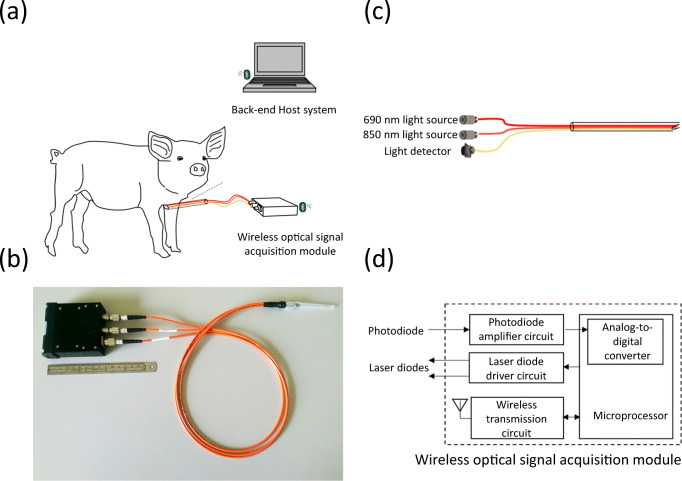
**a** Basic scheme. **b** Photograph of proposed tracheal recognition system. **c** Basic structure of optical probe. **d** Block diagram of wireless optical signal acquisition module

The fundamental principle of the proposed tracheal recognition system was based on the technique of near-infrared spectroscopy [15–20]. Figure 1 shows the basic scheme, photograph, structure, and block diagram of wireless optical signal acquisition module of the proposed tracheal recognition system. It mainly consisted of a needle-type optical probe and a wireless signal acquisition and control module. Here, the needle-type optical probe was designed to be inserted into the tissue easily, provide a dual-wavelength light source, and detect the light signal, penetrated through the tissue. The wireless signal acquisition and control module were designed to drive the light source of the optical probe, receive and digitize the reflected light signal, and transmit these signals to the back-end host system wirelessly. Finally, the back-end host system would calculate the relative oxy-hemoglobin (HbO_2_) and deoxy-hemoglobin (Hb) concentrations.

The needle-type optical probe mainly consisted of two laser diodes, a photodiode, optical fibers, and a needle structure. The optical fibers were inserted into the needle structure to transmit the incident light and the reflected light from these laser diodes and to the photodiode. The light, emitted from the optical probe, illuminates the tissue, and then, some photons would be absorbed or scattered by different components of the tissue. Different tissue components, such as skin, fat, muscle, and hemoglobin, provided different absorbing and scattering properties corresponding to different wavelengths [21–23]. Compared with skin, fat, and muscle providing higher absorbing properties, hemoglobin was one of major absorber in the wavelength range of red and near-infrared light (about 650~1300 nm). Then, by using the difference between the absorbing spectra of HbO_2_ and Hb, the relative HbO_2_ and Hb concentrations could be estimated by the optical density changes of dual-wavelength light via the modified Beer-Lambert law (mBLL) [24–27]. The change of optical density ∆OD(*λ*) corresponding to the specific wavelength *λ*, describing the attenuation of light caused from difference tissue components, could by expressed as:

1 $$ \Delta  \mathrm{OD}\left(\lambda \right)=-\log \frac{I_{\mathrm{ref}}\left(\lambda \right)}{I_{\mathrm{int}}\left(\lambda \right)}=\varepsilon CLB\left(\lambda \right) $$

where *I*_int_(*λ*), *I*_ref_(*λ*), and *B*(*λ*) denoted the intensities of incident light and light reflected from the tissue, and the differential path length factor corresponding to the wavelength *λ*, respectively, and the parameters *ε*, *C*, and *L* denoted the molar extinction coefficient, the molar concentration, and the distance between the light source and the detector, respectively. Because the absorption coefficient of hemoglobin was much greater than that of the other human tissues in the wavelength of near-infrared light, the absorbing effect of the other human tissues could be ignored and the optical density variation could be re-expressed by:

2 $$ \Delta  \mathrm{OD}\left(\lambda \right)=\left[{\varepsilon}_{\mathrm{Hb}{\mathrm{O}}_2}\times \left[\mathrm{Hb}{\mathrm{O}}_2\right]+{\varepsilon}_{\mathrm{Hb}}\times \left[\mathrm{Hb}\right]\right] LB\left(\lambda \right) $$

For dual-wavelengths *λ*_1_ and *λ*_2_, the relative HbO_2_ and Hb concentrations could then be solved by:

3 $$ \left[\mathrm{Hb}{\mathrm{O}}_2\right]=\left({\varepsilon}_{\mathrm{Hb}}\left({\lambda}_2\right)\times \frac{\Delta  \mathrm{OD}\left({\lambda}_1\right)}{B\left({\lambda}_1\right)}-{\varepsilon}_{\mathrm{Hb}}\left({\lambda}_1\right)\times \frac{\Delta  \mathrm{OD}\left({\lambda}_2\right)}{B\left({\lambda}_2\right)}\right)\times \frac{1}{\det \left(\left[\begin{array}{cc}{\varepsilon}_{\mathrm{Hb}{\mathrm{O}}_2}\left({\lambda}_1\right)& {\varepsilon}_{\mathrm{Hb}}\left({\lambda}_1\right)\\ {}{\varepsilon}_{\mathrm{Hb}{\mathrm{O}}_2}\left({\lambda}_2\right)& {\varepsilon}_{\mathrm{Hb}}\left({\lambda}_2\right)\end{array}\right]\right)}\times \frac{1}{L} $$

4 $$ \left[\mathrm{Hb}\right]=\left({\varepsilon}_{\mathrm{Hb}{\mathrm{O}}_2}\left({\lambda}_1\right)\times \frac{\Delta  \mathrm{OD}\left({\lambda}_2\right)}{B\left({\lambda}_2\right)}-{\varepsilon}_{\mathrm{Hb}{\mathrm{O}}_2}\left({\lambda}_2\right)\times \frac{\Delta  \mathrm{OD}\left({\lambda}_1\right)}{B\left({\lambda}_1\right)}\right)\times \frac{1}{\det \left(\left[\begin{array}{cc}{\varepsilon}_{\mathrm{Hb}{\mathrm{O}}_2}\left({\lambda}_1\right)& {\varepsilon}_{\mathrm{Hb}}\left({\lambda}_1\right)\\ {}{\varepsilon}_{\mathrm{Hb}{\mathrm{O}}_2}\left({\lambda}_2\right)& {\varepsilon}_{\mathrm{Hb}}\left({\lambda}_2\right)\end{array}\right]\right)}\times \frac{1}{L} $$

Here, det(.) was the determinant of the matrix. Here, a 690-nm laser diode (HL6738MG, Thorlabs, USA) and an 850-nm laser diode (L850P030, Thorlabs, USA) were used to provide dual-wavelength light source, because these two wavelengths straddled the isosbestic point of the HbO_2_ and Hb absorbing spectra. The photodiode (PD15-22C, EVERLIGHT, Taiwan) was used as the light detector.

The wireless signal acquisition and control module would drive these laser diodes to emit dual-wavelength light to the tissue, receive and digitize the reflected light signal, and then transmit these raw data to the back-end host system. Finally, the back-end host system would calculate the relative HbO_2_ concentration (HbO_2_) and the relative Hb concentration (Hb).

### Experimental design

**Fig. 2:**
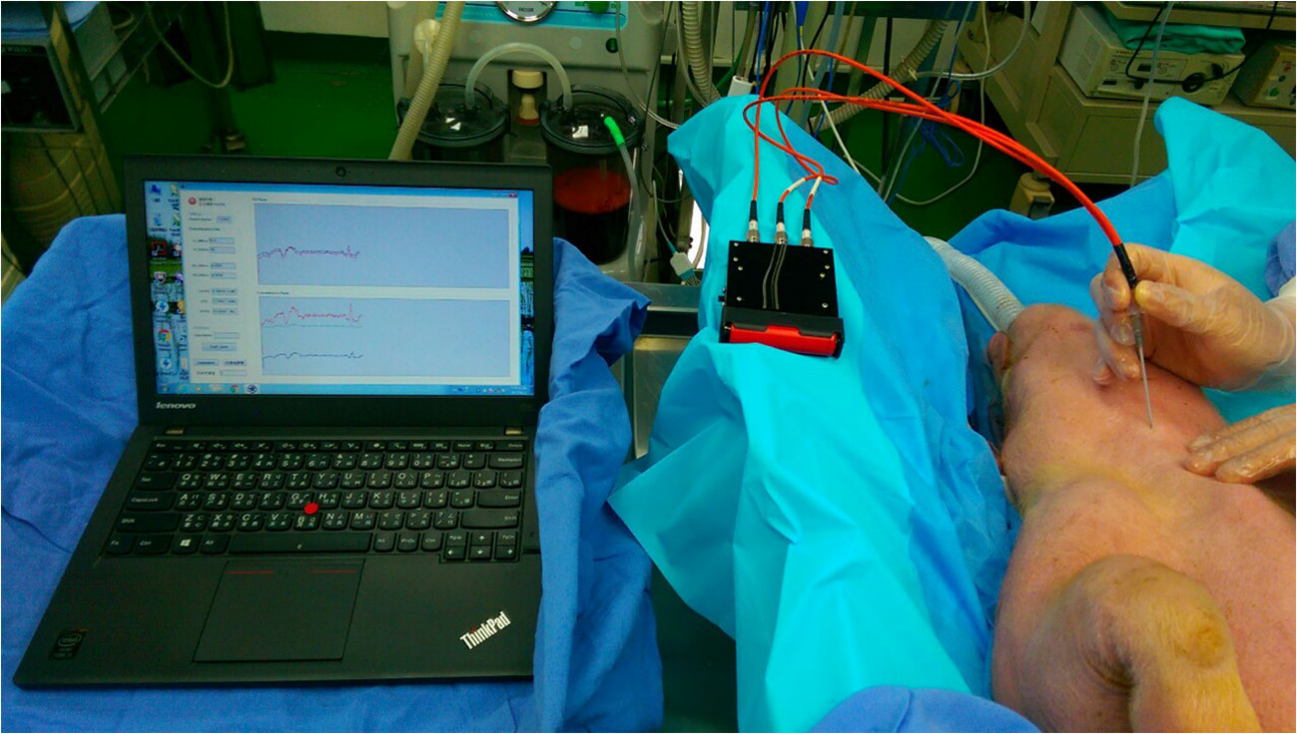
The demonstration of our proposed system in a fresh piglet. We use the program for analyzing the collecting signals

In this study, five duroc, Chinese native piglets with an average age 4 year old and average weight of 25 kg were used. Atropine 0.05 mg/kg and tiletamine-zolazepam 6 mg/kg were given intramuscularly for the induction of general anesthesia. These animals were intubated, ventilated, and then maintained with isoflurane (inhalation anesthetic). For the in vivo study, a vertical incision was made between the thyroid cartilage and the cricoid cartilage of the piglets. The necks of these piglets were dissected layer by layer until cricothyroid membrane was seen. Every visible layer from skin, fat, muscle, and cricothyroid membrane was separated. Then, we simulated the route of the needle in the angle of 45° and advanced the needle tip layer by layer as we performed the procedure of needle cricothyroidotomy. We used the embedded optical fiber needle emitted by lasers with 690-nm and 850-nm wavelengths layer by layer into different tissues. We stopped the needle tip in every tissue layer for about 10 s and collected these light signals on an oscilloscope and analyzed immediately. This tracheal recognition system was used to detect these reflected and scattered light signals from the skin to trachea. The internal jugular vein and common carotid artery of piglets were also dissected to simulate accidental vessel punctures. In Fig. 2, we used the program for analyzing the collecting signals. The program used for signal analysis was developed by ourselves based on Microsoft Visual C#, 2017, Microsoft, USA. We could see two analyzed data in the screen of the laptop. The upper portion of the screen was the change of optical density ∆OD(*λ*) and the lower portion was the estimated Hb concentration (blue color) and HbO_2_ concentrations (red color). We collected these signals and analyzed them immediately. The lag time of the analysis in the program was about 2 s. We could easily see the change of optical density, oxy-hemoglobin (HbO_2_) concentration, and deoxy-hemoglobin (Hb) in the laptop.

### Statistical methods

Serum Hb and HbO_2_ and optical density change corresponding to 690 nm and 850 nm were all repeatedly measured in various tissues in each of five individuals. Due to the small sample size, we used Kruskal-Wallis *H* test to compare differences of values across tissues at each time-point, respectively. We further performed a regression analysis with linear mixed model in order to simultaneously consider the random effect of repeated measures and fixed effect of tissue in relation to the observed values of optical density change and serum Hb and HbO_2_. Trachea group was set as the reference category in comparisons with optical density change, while vein and artery group was set as reference category in comparisons with Hb and HbO_2_, respectively. Statistical analyses in this study were generated using SAS/STAT software, version 9.4 of the SAS System for Windows—copyright © 2002–2012 by SAS Institute Inc., Cary, NC, USA.

## Results

**Fig. 3:**
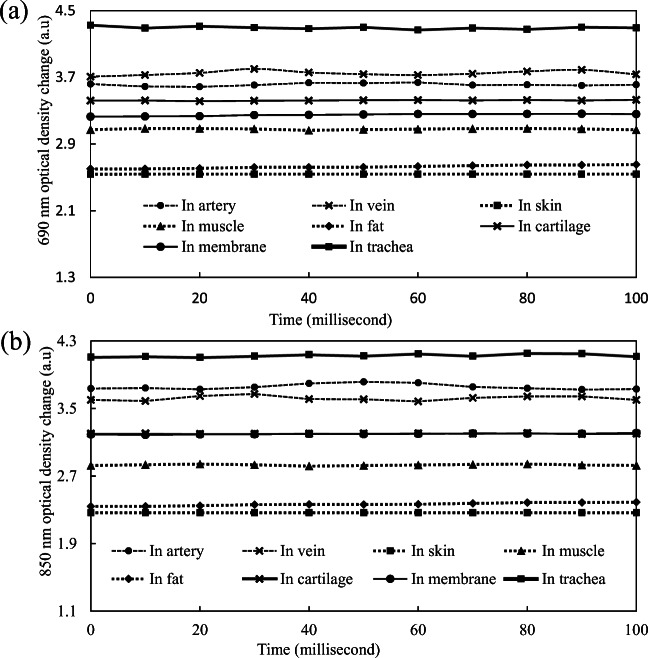
Change of optical density in different tissue components of pig neck corresponding to **a** 690-nm and **b** 850-nm wavelengths

The change of optical density corresponding to different wavelengths in different tissue components are obviously discriminable (Fig. 3a, b). For the wavelength of 690 nm, the change of the optical density in vein blood was higher than that in artery blood; however, corresponding to 850 nm, the change of the optical density in vein blood was lower than that in artery blood. Moreover, except for the tissue of trachea, the change of the optical densities in both artery and vein blood was higher than that of other tissue components. For both 690-nm and 850-nm wavelengths, the change of the optical density in the muscle group was relatively higher than that of the fat and skin group, and the change of the optical density in the skin group was the lowest. The change of the optical density in the trachea group was the highest, due to the very poor scattering and absorbing properties of air in the trachea. The optical density change of cartilage for a 690-nm wavelength was higher than that of the cricothyroid membrane group, but their optical density changes for the 850-nm wavelength were similar.

There were almost tenfold signal difference in the change of optical density (2.54 optical density change in the skin group and 3.42 optical density change in the cartilage group) corresponding to 690-nm wavelengths (Fig. 3a). There were also tenfold signal differences in the change of optical density (4.3 optical density change into the tracheal group and 3.25 optical density change in cricothyroid membrane group) corresponding to 690-nm wavelengths (Fig. 3a). In the artery and vein group, it showed that there were more light absorbed [28] and limited light was reflected and scattered. On the contrary, there were more light reflected and scattered in the skin and fat group. Moreover, the absorption coefficient of muscle is also higher than fat [29].

In Tables 1, 2, 3, and 4, we could see the results of statistical analyses including mean and standard errors for different tissue types. In Tables 1 and 2, both of the tracheal groups were set as reference category. The *p* values in every tissue in optical density change corresponding to 690 nm and 850 nm were all < 0.001. In Table 3, the vein group was set as reference category. In Table 4, the artery group was set as reference category. The *p* values in every tissue in Hb and HbO_2_ were also all < 0.001. The results were statistically significant.

**Table 1:** OD 690 nm

		Tissue																*p* value^1^
		Artery		Vein		Skin		Muscle		Fat		Cartilage		Membrane		Trachea		
Time	*N*	Mean	SE	Mean	SE	Mean	SE	Mean	SE	Mean	SE	Mean	SE	Mean	SE	Mean	SE	
1	5	3.62	0.0030	3.75	0.0131	2.54	0.0010	3.08	0.0017	2.62	0.0092	3.42	0.0018	3.24	0.0040	4.30	0.0089	< 0.001
2	5	3.61	0.0040	3.74	0.0045	2.54	0.0012	3.08	0.0031	2.62	0.0067	3.42	0.0014	3.25	0.0068	4.30	0.0047	< 0.001
3	5	3.61	0.0071	3.75	0.0047	2.54	0.0007	3.08	0.0044	2.63	0.0065	3.42	0.0019	3.25	0.0049	4.29	0.0086	< 0.001
4	5	3.60	0.0029	3.77	0.0104	2.53	0.0016	3.08	0.0032	2.63	0.0046	3.42	0.0031	3.25	0.0047	4.29	0.0034	< 0.001
5	5	3.61	0.0074	3.74	0.0102	2.54	0.0011	3.07	0.0033	2.63	0.0057	3.42	0.0010	3.25	0.0042	4.30	0.0089	< 0.001
6	5	3.62	0.0069	3.75	0.0055	2.54	0.0011	3.08	0.0031	2.62	0.0054	3.42	0.0012	3.25	0.0077	4.28	0.0070	< 0.001
7	5	3.61	0.0091	3.75	0.0127	2.54	0.0009	3.08	0.0030	2.63	0.0031	3.42	0.0022	3.26	0.0059	4.29	0.0077	< 0.001
8	5	3.61	0.0035	3.76	0.0145	2.54	0.0012	3.08	0.0034	2.64	0.0052	3.42	0.0025	3.24	0.0073	4.31	0.0067	< 0.001
9	5	3.62	0.0052	3.75	0.0084	2.54	0.0026	3.08	0.0031	2.62	0.0073	3.43	0.0019	3.26	0.0053	4.30	0.0093	< 0.001
10	4	3.61	0.0025	3.76	0.0114	2.54	0.0015	3.08	0.0054	2.63	0.0091	3.42	0.0018	3.25	0.0033	4.29	0.0025	< 0.001
11	1	3.61	.	3.74	.	2.54	.	3.07	.	2.65	.	3.43	.	3.26	.	4.29	.	
Total	50	3.61	0.0018	3.75	0.0031	2.54	0.0004	3.08	0.0010	2.63	0.0021	3.42	0.0007	3.25	0.0017	4.30	0.0023	
*p* value^2^		< 0.001		< 0.001		< 0.001		< 0.001		< 0.001		< 0.001		< 0.001		Ref.		

*Ref.*, reference category

^1^Kruskal-Wallis *H* test

^2^Fixed effect of tissue was examined in a linear mixed model considering repeated measures as a random effect

**Table 2:** OD 850 nm

		Tissue																*p* value^1^
		Artery		Vein		Skin		Muscle		Fat		Cartilage		Membrane		Trachea		
Time	*N*	Mean	SE	Mean	SE	Mean	SE	Mean	SE	Mean	SE	Mean	SE	Mean	SE	Mean	SE	
1	5	3.75	0.0103	3.61	0.0047	2.26	0.0024	2.83	0.0043	2.36	0.0048	3.20	0.0004	3.20	0.0014	4.12	0.0032	< 0.001
2	5	3.75	0.0058	3.62	0.0099	2.27	0.0007	2.83	0.0063	2.36	0.0069	3.20	0.0012	3.20	0.0026	4.12	0.0025	< 0.001
3	5	3.75	0.0087	3.62	0.0076	2.26	0.0006	2.84	0.0031	2.36	0.0034	3.20	0.0025	3.19	0.0024	4.12	0.0086	< 0.001
4	5	3.75	0.0133	3.63	0.0141	2.26	0.0013	2.83	0.0014	2.37	0.0051	3.20	0.0024	3.20	0.0024	4.13	0.0052	< 0.001
5	5	3.76	0.0104	3.61	0.0041	2.27	0.0016	2.82	0.0079	2.36	0.0042	3.20	0.0010	3.20	0.0017	4.13	0.0039	< 0.001
6	5	3.77	0.0140	3.63	0.0132	2.27	0.0018	2.83	0.0026	2.36	0.0032	3.20	0.0008	3.20	0.0003	4.12	0.0014	< 0.001
7	5	3.76	0.0118	3.62	0.0088	2.27	0.0012	2.83	0.0029	2.36	0.0055	3.20	0.0024	3.20	0.0011	4.14	0.0083	< 0.001
8	5	3.76	0.0087	3.62	0.0061	2.26	0.0022	2.83	0.0059	2.37	0.0051	3.20	0.0026	3.20	0.0033	4.12	0.0079	< 0.001
9	5	3.76	0.0073	3.62	0.0074	2.27	0.0012	2.84	0.0032	2.37	0.0072	3.20	0.0018	3.20	0.0013	4.14	0.0077	< 0.001
10	4	3.75	0.0107	3.63	0.0089	2.26	0.0014	2.83	0.0017	2.37	0.0041	3.20	0.0012	3.20	0.0007	4.14	0.0069	< 0.001
11	1	3.73	.	3.60	.	2.27	.	2.83	.	2.39	.	3.20	.	3.21	.	4.12	.	
Total	50	3.76	0.0031	3.62	0.0027	2.27	0.0005	2.83	0.0014	2.37	0.0018	3.20	0.0006	3.20	0.0007	4.13	0.0020	
*p* value^2^		< 0.001		< 0.001		< 0.001		< 0.001		< 0.001		< 0.001		< 0.001		Ref.		

*Ref.*, reference category

^1^Kruskal-Wallis *H* test

^2^Fixed effect of tissue was examined in a linear mixed model considering repeated measures as a random effect

**Table 3:** Hb

		Tissue														*p* value^1^
		Artery		Vein		Skin		Muscle		Fat		Cartilage		Membrane		
Time	*N*	Mean	SE	Mean	SE	Mean	SE	Mean	SE	Mean	SE	Mean	SE	Mean	SE	
1	5	2.74	0.0074	2.93	0.0100	2.04	0.0002	2.45	0.0043	2.09	0.0036	2.71	0.0013	2.52	0.0049	< 0.001
2	5	2.75	0.0059	2.93	0.0077	2.04	0.0002	2.45	0.0018	2.10	0.0060	2.70	0.0013	2.52	0.0041	< 0.001
3	5	2.75	0.0058	2.93	0.0063	2.04	0.0001	2.44	0.0022	2.10	0.0079	2.70	0.0023	2.53	0.0050	< 0.001
4	5	2.74	0.0047	2.94	0.0086	2.04	0.0002	2.45	0.0068	2.10	0.0062	2.71	0.0014	2.52	0.0039	< 0.001
5	5	2.75	0.0064	2.92	0.0053	2.04	0.0003	2.44	0.0038	2.10	0.0042	2.70	0.0013	2.52	0.0074	< 0.001
6	5	2.74	0.0042	2.93	0.0043	2.04	0.0001	2.45	0.0024	2.09	0.0032	2.71	0.0033	2.51	0.0055	< 0.001
7	5	2.75	0.0038	2.93	0.0047	2.04	0.0002	2.45	0.0020	2.10	0.0030	2.71	0.0020	2.53	0.0029	< 0.001
8	5	2.75	0.0045	2.94	0.0097	2.04	0.0002	2.45	0.0071	2.11	0.0052	2.70	0.0057	2.53	0.0090	< 0.001
9	5	2.74	0.0093	2.92	0.0113	2.04	0.0001	2.45	0.0024	2.10	0.0053	2.70	0.0038	2.52	0.0040	< 0.001
10	4	2.74	0.0039	2.93	0.0147	2.04	0.0001	2.44	0.0065	2.12	0.0041	2.70	0.0024	2.53	0.0020	< 0.001
11	1	2.75	.	2.92	.	2.04	.	2.44	.	2.12	.	2.71	.	2.53	.	
Total	50	2.75	0.0018	2.93	0.0026	2.04	0.0001	2.45	0.0013	2.10	0.0018	2.70	0.0009	2.52	0.0017	
*p* value^2^		< 0.001		Ref.		< 0.001		< 0.001		< 0.001		< 0.001		< 0.001		

*Ref.*, reference category

^1^Kruskal-Wallis *H* test

^2^Fixed effect of tissue was examined in a linear mixed model considering repeated measures as a random effect

**Table 4:** HbO_2_

		Tissue														*p* value^1^
		Artery		Vein		Skin		Muscle		Fat		Cartilage		Membrane		
Time	*N*	Mean	SE	Mean	SE	Mean	SE	Mean	SE	Mean	SE	Mean	SE	Mean	SE	
1	5	5.50	0.0209	5.11	0.0149	3.05	0.0007	3.88	0.0028	3.19	0.0061	4.43	0.0002	4.55	0.0050	< 0.001
2	5	5.47	0.0105	5.08	0.0127	3.05	0.0014	3.88	0.0030	3.20	0.0107	4.43	0.0015	4.54	0.0022	< 0.001
3	5	5.46	0.0125	5.09	0.0157	3.05	0.0004	3.88	0.0052	3.20	0.0117	4.43	0.0003	4.53	0.0067	< 0.001
4	5	5.49	0.0255	5.11	0.0157	3.05	0.0009	3.88	0.0033	3.19	0.0066	4.43	0.0013	4.54	0.0024	< 0.001
5	5	5.48	0.0183	5.09	0.0086	3.05	0.0009	3.88	0.0042	3.20	0.0070	4.43	0.0004	4.54	0.0057	< 0.001
6	5	5.48	0.0317	5.08	0.0077	3.05	0.0002	3.89	0.0055	3.21	0.0049	4.43	0.0009	4.53	0.0025	< 0.001
7	5	5.50	0.0176	5.08	0.0133	3.05	0.0013	3.88	0.0038	3.21	0.0118	4.43	0.0021	4.55	0.0073	< 0.001
8	5	5.45	0.0079	5.10	0.0140	3.05	0.0005	3.88	0.0042	3.21	0.0070	4.43	0.0018	4.54	0.0019	< 0.001
9	5	5.47	0.0086	5.09	0.0098	3.05	0.0010	3.89	0.0034	3.21	0.0072	4.43	0.0021	4.54	0.0057	< 0.001
10	4	5.47	0.0255	5.09	0.0059	3.05	0.0014	3.88	0.0025	3.21	0.0109	4.43	0.0025	4.53	0.0063	< 0.001
11	1	5.42	.	5.06	.	3.05	.	3.87	.	3.24	.	4.42	.	4.56	.	
Total	50	5.47	0.0059	5.09	0.0039	3.05	0.0003	3.88	0.0012	3.20	0.0027	4.43	0.0005	4.54	0.0016	
*p* value^2^		Ref.		< 0.001		< 0.001		< 0.001		< 0.001		< 0.001		< 0.001		

*Ref.*, reference category

^1^Kruskal-Wallis *H* test

^2^Fixed effect of tissue was examined in a linear mixed model considering repeated measures as a random effect

Under the presumption that type I error was set as 0.08 and standard deviation was 0.05, we targeted to detect a clinical significant difference of 0.2 with a statistical power of 90%; the sample size per group in comparisons of OD 650 nm, OD 850 nm, Hb, and HbO_2_ between any two tissues was expected to more than 4. The small sample size needed in two independent sample comparisons was due to the very high precision (small variation) in our measurements.

## Discussions

Our optical technology could be used to assist the procedure of needle cricothyroidotomy. The technique was easily adapted for use in a standard 16-gauge intravenous needle catheter (Fig. 1). The procedure of needle cricothyroidotomy using this optical technique was almost the same as traditional needle cricothyroidotomy, except there was a fiber bundle extended from the end stand of the intravenous needle catheter, and the information for recognizing the trachea was obtained by observing the signal change from an oscilloscope. Analysis of dual-wavelength optical data in the swine model presented that different tissues had unique and significant optical characteristics. This proposed system might provide us information in the needle tip and it could accurately and efficiently guide the needle tip when we performed the procedures.

There are several typical near-infrared spectroscopy (NIRS) applications of medical and physiological diagnostics and researches including pulse oximetry, blood sugar, functional neuroimaging, brain computer interface, urology (bladder contraction), and neurology (neurovascular coupling) [30–35]. The near-infrared light can provide a better penetrating depth in the living tissue. Measuring the blood flow information in the regional tissue including skin, fat, and muscle can be obtained with the aid of NIRS [36–40]. With the aid of the fundamental principle of near-infrared spectroscopy, this novel optic fiber technique might be helpful for guiding our needle tip into the trachea. NIRS can provide the real-time perspective monitoring of the tissue microcirculation, and it can also guide the needle tip into the trachea layer by layer. These optic fiber bundle with the dual-wavelength light source and light detector were embedded into the intravenous needle catheter. This proposed system could exactly help us to recognize different tissues in the swine model.

The structures of different tissues were histologically distinct. Furthermore, the microcirculation in different tissue was also different. These might result in the different light reflected signals. We could also recognize the interesting point that the least light signal was detected in the tracheal group from these dual-wavelengths of 690- and 850-nm light systems. When the light pass through the air in the hollow trachea, there was almost no reflected and no scattered light signal received by the detector.

**Fig. 4:**
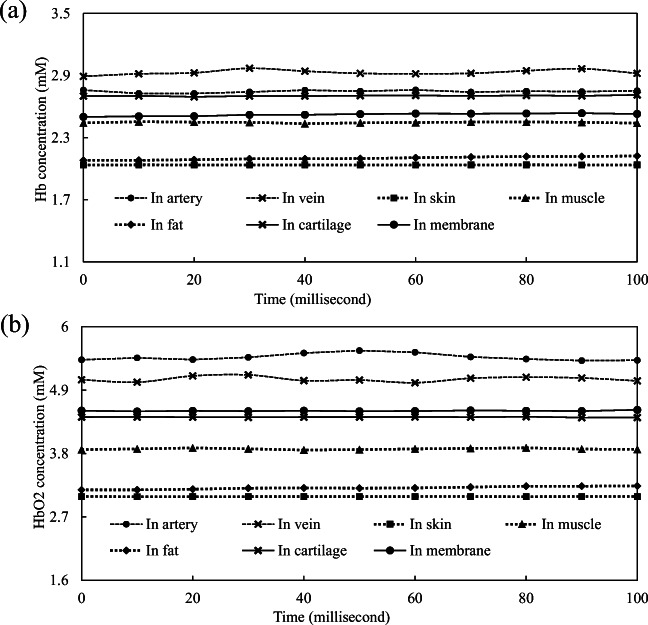
**a** Estimated Hb concentration and **b** HbO_2_ concentrations in different tissue components of pig neck

The estimated Hb and HbO_2_ concentrations in different tissue components are shown in Fig. 4a and b. The HbO_2_ concentration in artery blood was obviously higher than that in vein blood, and the Hb concentration in vein blood was relatively higher. Moreover, the HbO_2_ and Hb concentrations in both artery and vein blood were higher than that of fat, skin, cartilage, and cricothyroid membrane. Because the tissue of the trachea is a tubular hollow structure, light signals were rarely reflected and scatted. Why were the estimated HbO_2_ and Hb concentrations in trachea group highest? In theory, the hemoglobin was fewer in the trachea group than in the artery group or in the vein group. HbO_2_ and Hb concentrations were calculated according to optical absorption of hemoglobin. Hemoglobin is one of the major absorbers. We calculated higher HbO_2_ and Hb concentrations when more light was absorbed and limited light was reflected and scattered. In the trachea group, there was rarest light reflected and scattered. This phenomenon resulted in these unique significant optical differences. Subcutaneous emphysema or subcutaneous hematoma may interfer the signal collection. Hemoglobin was the main absorber of photons according to the modified Beer-Lambert law. Therefore, we avoided these situations in our study.

**Fig. 5:**
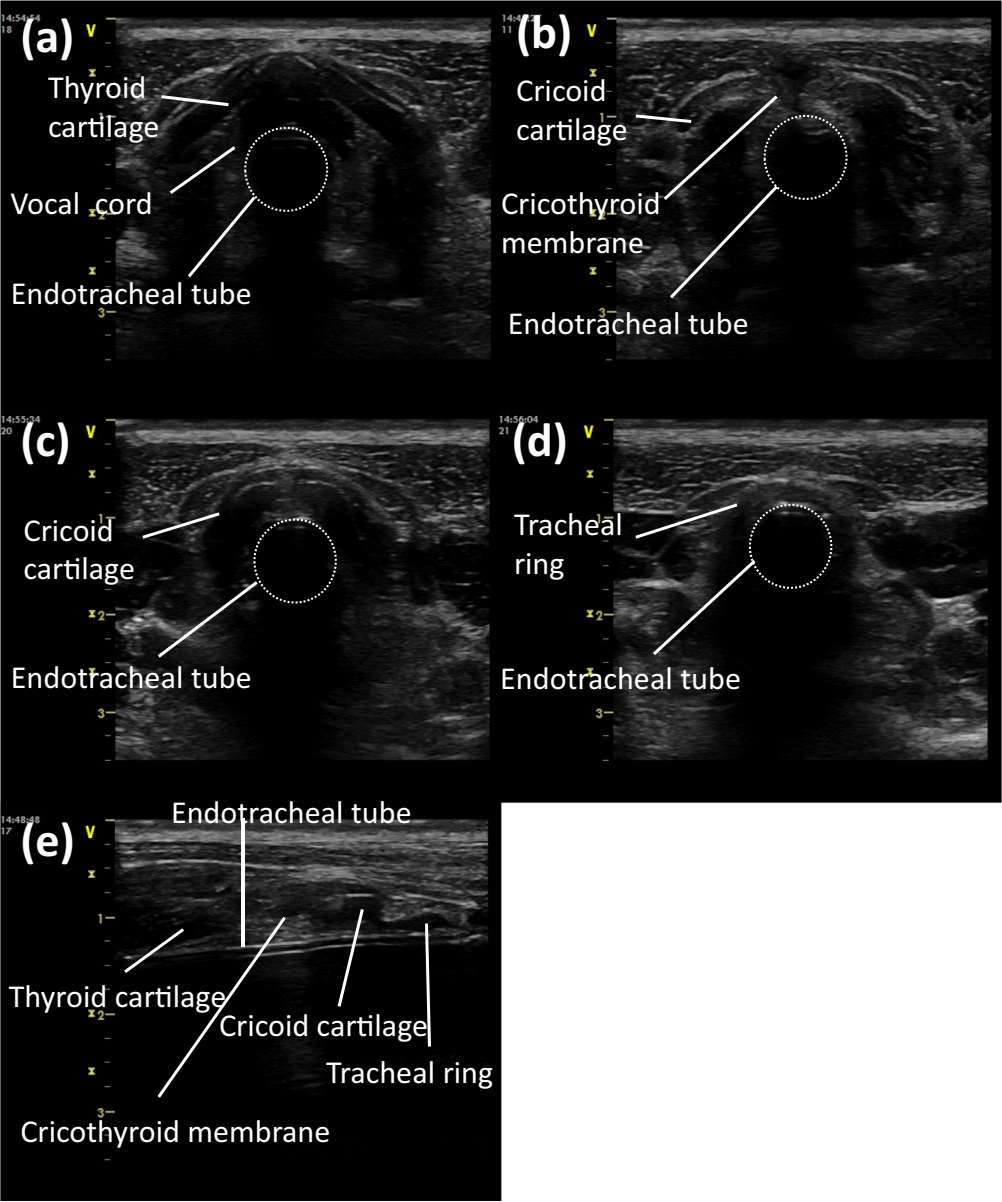
Sonograms (transverse views) in the piglets. **a** The thyroid cartilage level. **b** The cricothyroid membrane level. **c** The cricoid cartilage level. **d** The tracheal ring level. **e** Sonograms (longitudinal views) of the larynx and trachea in the piglets

Needle cricothyroidotomy with percutaneous transtracheal ventilation emerged as the invasive rescue method of choice because it provided oxygenation as well as clearance of carbon dioxide. The catheter size (13- to 16-gauge) does not appear to substantially affect gas flow rates when using wall outlet oxygen because gas flows in a turbulent fashion under high pressure through these narrow catheters [41]. The risk of pulmonary aspiration in needle cricothyroidotomy and percutaneous transtracheal ventilation was relative low compared with a cuffed tracheal tube. The flow of gas up the airway aids in the expulsion of secretions, minimizing the need for suction and preventing pulmonary aspiration [42]. The cricothyroid membrane, as the name implies, is bound by the cricoid cartilage inferiorly and the thyroid cartilage superiorly. Although ultrasound-guided cricothyroidotomy may be helpful, the needle tip may be out of view in an out-of-plane configuration [11, 12, 43]. The thyroid cartilage is one of the important landmarks for cricothyroidotomy as well as transtracheal injection for topically anesthetizing the tracheal mucosa. In our study, we also used ultrasound to obtain sonographic images in these piglets (Fig. 5), but it was difficult for us to identify the needle tip in an out-of plane approach. The depth of cricothyroid membrane in these piglets was about 1 cm, and it was much deeper than normal human being. Our proposed system might be helpful to guide the needle tip in the obese patients with increased neck circumference [44]. The largest cartilage of the larynx appeared as a thin inverted V-shaped hypoechoic structure in a transverse sonographic view (Fig. 5a). The cricoid cartilage (Fig. 5c) is another important landmark to identify before performing cricothyroidotomy or transtracheal injection through the cricothyroid membrane. The gradual change of the cricothyroid membrane (Fig. 5b) to the cricoid arch formation anteriorly could be displayed in a transverse sonographic view from cephalad to caudad. The cricoid cartilage appeared more “cuboid” in shape. Compared with the cricoid cartilage, the tracheal ring (Fig. 5d) was shown more small and round. In a sonographic longitudinal view of the larynx and trachea (Fig. 5e), the key anatomic landmarks are (from cephalad to caudad) the thyroid cartilage, the cricothyroid membrane, the cricoid cartilage, and the tracheal rings. The clinician should place the largest intravenous needle catheter possible using this limited information and palpation of the cricothyroid membrane as a guide. There are some differences in human and swine models, but in our study, we provided another feasible technique to perform needle cricothyroidotomy procedure. We collected different tissue optical parameters to be calculated, and we used the program for analyzing immediately. These unique and real-time optical data could provide us information to perform the needle cricothyroidotomy in a swine model.

Limitations of our present study included the subcutaneous emphysema, subcutaneous hematoma, and the small sample size. We avoided subcutaneous emphysema and subcutaneous hematoma when we collected data. Another limitation of our proposed system is that the direction of the beveled needle influenced the results while approaching different tissue layer, so we simulated the route of the needle in the angle of 45° in our study. The amount of the information obtained by using our technique may be limited, but our study provided a helpful evidence to assist needle cricothyroidotomy in swine model. The technique will require further testing to compare the procedural time and accuracy for different cricothyroidotomy procedures (ultrasonography versus NIRS guidance). Finally, there seems to be a different but quick learning curve similar to other cricothyroidotomy procedures.

## Conclusions

This is the first study to introduce a novel and unique optical technique to recognize the trachea. This system provides the operator with real-time information that can be displayed as a visual electronic signal to assist needle cricothyroidotomy.
